# One risk assessment for genetically modified plants

**DOI:** 10.3389/fbioe.2025.1619857

**Published:** 2025-07-18

**Authors:** Muffy Koch, Jaylee DeMond, Matthew G. Pence, Elena A. Schaefer, Gary Rudgers

**Affiliations:** Simplot Plant Sciences, J. R. Simplot Company, Boise, ID, United States

**Keywords:** GMO, low risk, regulatory efficiency, RNA interference, vegetative crops, cisgenes

## Abstract

With over 30 years’ experience conducting risk assessments for genetically modified (GM) plants, regulatory agencies that review the safety of GM plants understand the potential food, feed, and environmental risks associated with these products. This vast regulatory experience is underutilized when risk assessments for GM plants are repeated on a per-country basis. The redundancy in country-by-country reviews of the same GM plants places a disproportionate regulatory burden on developers and strains limited government resources for conducting safety reviews. Requiring repeated, multi-country risk assessments to obtain food and feed import permits or cultivation permits for GM plants is unnecessary as repeated assessments do not change the safety and associated risks of already approved products. To avoid redundancies in the regulation of GM plants, we propose adoption of one, global risk assessment for food, feed, and environmental release carried out to international standards. Our proposed model for one global risk assessment encourages the sharing of food, feed and environmental risk assessment summaries between countries while maintaining national approvals for GM plants. Steps towards a streamlined and efficient review process for GM plants are discussed, including implementing a global, forward-looking approval process that eliminates repetitive risk assessments and re-reviews of low-risk traits. Harmonization of risk assessment is an achievable goal that would accelerate regulatory approvals and enable broader access to the benefits of GM plants which are currently only available to some countries.

## Introduction

As we enter the fourth decade of genetically modified (GM) plants being approved and used internationally, regulatory experience with environmental risk assessments for cultivation approvals, and food safety risk assessments for food and feed approvals should be applied to remove unnecessary pre-market regulatory requirements and broaden market access for safe and beneficial products of biotechnology. Global harmonization of risk assessments for GM plants is an achievable goal that would accelerate regulatory approvals and expand access and deployment of safe and beneficial agricultural products with the potential to secure the food needs of an increasing world population (Koch et al., 2024). Streamlining the regulatory process for GM plants will improve equitable access to the technology for small developers, the public sector, farmers, and consumers. The Organization for Economic Cooperation and Development member countries have been working on global harmonization of environment, food and feed safety reviews for many years and have published a range of consensus reports to guide countries as they set up review standards for GM plants (OECD, 2006; 2015). In addition, others have proposed a core set of studies to support food and feed safety assessments globally (Waters et al., 2021) and encouraged the streamlining of data requirements for environmental risk assessments (Anderson et al., 2021). We expand on these ideas with a model for implementing shared risk assessments and proactive approvals.

Countries with regulatory frameworks that are actively reviewing and making timely decisions on the food and feed safety of GM organisms generally follow the CODEX CAC/GL 45-2003 guidelines (FAO/WHO, 2009). As part of the review process, regulators consider safety data collected on new GM plants and identify potential hazards associated with consumption of food and feed products made from these plants. GM plants are compared to conventional plants that already have a history of safe use. Food and feed biosafety reviews take into consideration how the composition of a new event (event = a unique plant-trait combination) may impact the nutrition of products derived from it, the potential for changes in toxicity or allergenicity, changes to levels of endogenous antinutrients, and whether the modification changes how the plant will be used for food and feed. For any identified potential hazard, reviewers assess the likelihood of harm, the consequences should harm occur, mechanisms to manage risk, and whether the overall risk is acceptable in terms of a country’s national protection goals.

Environmental safety reviews focus on how a new trait could potentially impact a GM plant’s biology compared to conventional plants, and evaluate changes to invasiveness, weediness, persistence, gene flow to sexually compatible plants, and impacts on non-target organisms (OGTR, 2013). For identified, potential environmental hazards, a similar risk assessment as for food and feed considers the likelihood of harm, the consequences should harm occur, mechanisms to manage risk, and whether the overall risk is acceptable in terms of national protection goals.

Experience with safety assessments of GM plants has confirmed that potential risks to the environment or to human or animal health are identifiable and manageable in approved plants (Radin, 2003; Sanchez, 2015). At least 645 GM events have been reviewed for cultivation, food and feed safety in 46 countries (ISAAA, 2024). In most instances, the initial environmental, food and feed safety reviews were completed in the country where the plant was developed, first introduced to farmers, and placed on the market. In an unnecessary abundance of caution, the same food and feed safety reviews for GM plants are repeated in many countries where food and feed from the GM plant will be imported. Re-review is unnecessary to establish that an already approved GM plant is safe for human and animal consumption. Experience tells us that a single, well-constructed review is able to identify potential risks in a newly developed GM plant.

Once the safety has been determined for an approved plant-trait combination, repeated reviews are not needed and are costly for developers and regulators, delay deployment of previously approved GM plants, restrict developers, and limit farmers’ access to improved planting material. An industry study determined that the cost of developing and commercializing a new GM plant was US$ 115 million and 38% of this cost, US$ 43 million, was for regulatory approvals (AgbioInvestor, 2022). For agencies that charge for regulatory reviews, the fees can be from US$ 5,000 to US$ 450,000, but costs agencies accrue to conduct GM risk assessments are likely higher.

We discuss ways to reduce this unnecessary regulatory burden and provide a model for moving ahead with a more streamlined, equally safe and efficient one global risk review of GM plants. In addition, examples are provided of low-risk GM plants where one environmental and one food and feed assessment are also sufficient for cultivation approval in many release environments and for human and animal consumption.

## One global food and feed risk assessment

The data requirements for food and feed risk assessments are clearly defined and universal (Waters et al., 2021), therefore, once food safety has been determined, a global risk assessment summary could be used to eliminate additional food and feed reviews and inform food and feed approvals in all other countries.

Section 3 in Annex 3 of the CODEX guidelines (FAO/WHO, 2009) encourages member states to share risk assessment and safety information to facilitate food import approvals in other countries (FAO/WHO, 2009). Vietnam took an early step towards a global food and feed safety approval when they introduced a 2014 policy to accept the food safety of a GM plant that had food safety approval from five developed countries (Ministry of Agriculture and Rural Development, 2014). In 2013, Health Canada (HC) and Food Standards Australia and New Zealand (FSANZ) began collaborating on food safety risk assessments to streamline the review process for new GM plants in acknowledgement that food safety in one country is applicable in other countries (FSANZ, 2024). Bangladesh, Bhutan, India and Sri Lanka have been in discussion since 2020 about harmonization of food and feed safety assessments across these four countries (AFSI, 2025).

A review of approvals listed in the International Service for the Acquisition of Agri-biotech Applications (ISAAA) GMO Approval Database indicates that many events have had multiple reviews in multiple countries and by many agencies (ISAAA, 2024). After food safety reviews of 645 GM events in 46 countries, the learnings from these reviews need to be applied to simplify and modernize global reviews for food safety. For example, the maize event, MON810, was approved early in the development of GM crops and has 25 food approvals, 20 feed approvals, and 14 cultivation approvals listed in the ISAAA database (ISAAA, 2025), in addition to many more approvals as a stacked event that was bred together with other approved maize events. We cannot say whether the subsequent approvals identified unique country-specific safety concerns, but ultimately the many reviews did not change the first findings that the GM maize was safe for cultivation, food and feed use.

There may be instances where additional potential hazards are identified when the initial food and feed risk assessment is reviewed in another country. In this case the agency can focus their risk assessment on this concern, considering the pathway to harm, likelihood, consequences and risk management options for the new hazard without the need to reassess the previously reviewed hazards. For example, food consumption patterns or novelty of a food may require additional review in some countries. This happened in the 1990s in South Africa with review of the MON810 maize event for general release approval. United States consumption data do not accurately reflect consumption in South Africa where maize is the staple food and consumed at much higher levels. United States maize consumption is estimated at 2.85 g/person/day (EPA, 2014), nearly eight times lower than the South African *per capita* consumption of 222 g/person/day (Ranum et al., 2014). The scientific review committee asked the applicant to provide exposure data for the South African population. Importantly, in this example the review committee identified the potential higher exposure that was not addressed in the application and asked for specific data to address that gap. This expedited the food review by focusing only on new potential hazards.

In our experience, following the approval of nine potato events in the United States, subsequent food and feed safety reviews in other countries have not identified any additional potential hazards (Table 1). The pSIM1278 construct is present in eight potato events. Collectively, the safety of the traits in pSIM1278 has been independently assessed and approved in 54 regulatory reviews by 10 agencies in nine countries (United States: Food and Drug Administration (FDA); Canada: HC and Canadian Food Inspection Agency (CFIA); Mexico: Federal Commission for the Protection against Sanitary Risk; Japan: Ministry of Health Labor and Welfare, Ministry of Agriculture, Forestry, and Fisheries; Philippines: Bureau of Plant Industry; Australia/New Zealand: FSANZ; Malaysia: Department of Biosafety; Singapore: Singapore Food Agency). Since the initial food and feed safety assessment by the FDA in 2013, none of the subsequent 53 reviews identified any new potential hazards or raised additional safety concerns regarding these traits in potatoes. These cumulative evaluations have consistently affirmed the safety of the traits and have not added or subtracted from the safety of the events. These events included five potato varieties, six different traits, and one selection strategy. Even with the diversity in genomic backgrounds and traits, no new safety concerns were identified in any of the subsequent reviews undertaken by food importing countries.

**TABLE 1:** Global regulatory approvals for GM potato events.

Construct	Event name	Global approvals per event^a^		Global reviews per construct
		Environment	Food/Feed	
pSIM1278	E12	2	9	32
	F10	2	5	
	J3	2	5	
	V11	2	5	
pSIM1678 (+pSIM1278)	X17	3	10	42
	Y9	3	10	
	Z6	3	6	
	W8	3	4	
pSIM4363	BG25	1	3	4
Totals		21	57	78

^a^ All reviewed and approved for food and feed. Eight approved for cultivation in United States and Canada.

This is evidence that the repeated food safety reviews by importing countries do not increase the safety of the products. Instead, these redundant reviews confirmed the functionality of the initial review that was based on the CODEX guidelines. For potatoes, as these same traits are transformed into additional potato varieties, the current country-by-country review process will generate even more redundant risk assessments until a more science based approach to risk assessment is adopted globally.

## One global environmental risk assessment

Data requirements for environmental risk assessments are defined clearly and are transportable across countries (Anderson et al., 2021; M. Bachman et al., 2021). While countries differ in their geographical locations and environments, it is not necessarily the case that one global environmental risk assessment would have little value due to environmental differences. Findings of invasiveness, weediness, gene flow, persistence, and impact on non-target organisms in the first country can be used to inform risk assessment in subsequent countries. The risk assessment findings from the initial review will need to be reviewed in subsequent countries, and this can be done with the help of local crop, trait and biodiversity experts without the need for in-country studies or additional data from developers/applicants. Using problem formulation methodology, these experts can identify whether novel pathways to harm are likely and focus any additional assessments on clearly defined, country-specific risk hypotheses that were not addressed in the initial review (Wolt et al., 2010). Importantly, while plant performance is environment-specific, the performance of a plant is not a safety consideration in a regulatory approval.

In cases where regulatory agencies determine traits introduced into GM plants are low risk, one global risk assessment for environmental release should also be feasible. Three decades of environmental safety review of GM plants confirm that some categories of GM events are low risk across many environments.

## Low-risk traits

Three categories of introduced traits have developed a history of safe use in edible crops, have been applied widely in commercial GM plants, and are recognized as safe by regulatory authorities. Examples of low-risk traits include those conferred using RNA interference (RNAi), retransformation of additional varieties of approved vegetatively propagated GM plants, and the use of cisgenic traits that are inserted into sexually compatible plants. These three groups of GM plants are discussed in more detail.

### RNA interference

RNA interference involves the processing of endogenous double stranded RNA (dsRNA) into small RNA (e.g., miRNA, siRNA, etc.) to regulate gene expression in cells. This mechanism is common in plants, insects, fungi, nematodes, and animals (Shabalina and Koonin, 2008; Wilson and Doudna, 2013). Analysis of plant transcriptomes indicates that dsRNA are abundant in plants, with over eight million long dsRNA predicted in just five conventional crops: corn, soy, rice, lettuce, and tomato (Jensen et al., 2013). These naturally abundant dsRNA are processed by the plant into approximately 29 million unique small RNA with 100% complementarity to over 1,500 human transcripts (Ivashuta et al., 2009; Jensen et al., 2013; Frizzi et al., 2014). A diverse population of small RNA has been identified in conventional potato tubers (Zhang et al., 2013). This enormous diversity of endogenous small RNA is consumed regularly by humans and animals without incident. While there have been attempts to demonstrate that small RNAs from plants are capable of regulating human gene expression when humans are exposed to RNA from the consumption of plant-based foods, these findings are based on poor experimental design or misinterpretation of results (Pastrello et al., 2016).

Based on the universal presence of nucleic acids in the cells of every living organism, including every plant, animal and microbe used for food or feed, there is a long history of safe consumption of RNA and DNA. In 1998, the FDA reached a conclusion, stating that nucleic acids are “generally recognized as safe” (57 Fed Reg. 22984, 22990, 29 May 1992), and noting that “Introduced nucleic acids, in and of themselves, do not raise safety concerns. Thus, for example, the introduction of a gene encoding an anti-sense RNA would not raise concerns about either the gene or the anti-sense RNA. Any safety considerations would focus on the intended effects of the anti-sense RNA” (FDA, 1992). In 2001, the United States EPA established an exemption from the requirement for a tolerance for residues of nucleic acids (40 C.F.R. 174.507) under the Federal Food, Drug, and Cosmetic Act, noting that “nucleic acids are ubiquitous in all forms of life, have always been present in human and domestic animal food, and are not known to cause any adverse health effects when consumed as part of food” (66 Fed. Reg. 37817, 19 July 2001). The GRAS status of RNA has been relied on by United States Department of Agriculture (USDA) in regulatory decisions (USDA, 2006; 2011). In 2013, Food Standards Australia New Zealand, which evaluates food safety requirements from biotechnology foods, stated, “There is no scientific basis for suggesting that small dsRNA present in some GM foods have different properties or pose a greater risk than those already naturally abundant in conventional foods” (FSANZ, 2013). The history of safe use of nucleic acids, including small RNA, was based on its presence and safe use in all plants, including all plants that are used for food by humans.

Through naturally occurring evolutionary processes during plant adaptation and the use of traditional breeding practices, many plants and conventional cultivars contain inverted repeats because of genetic rearrangements. These are expressed as dsRNA, interact with Dicer to produce siRNA, and regulate expression of endogenous genes using RNA interference (Parrott et al., 2010; Petrick et al., 2013). Soybean seed color, maize stalk color, and rice protein content are examples of RNAi-based traits obtained through conventional breeding (Kusaba et al., 2003; Tuteja et al., 2004; Della Vedova et al., 2005).

Food and feed derived from approved GM plants using RNAi-based gene regulation is expected to be as safe for consumption as food and feed derived from conventional breeding (Petrick et al., 2013). From 1994 to 2022, over 30 RNAi-based events were approved by regulators for food, feed, or cultivation in alfalfa, apple, bean, maize, papaya, plum, potato, soybean, squash, and tomato. Approximately 242 (ISAAA, 2022) food and feed approvals exist in over 20 countries for RNAi-based events (ISAAA, 2022). These risk assessment decisions demonstrate that products with RNAi-based traits are considered safe for human and animal consumption, and subsequent safe consumption of these events in food and feed confirms that these findings are correct. Based on 242 food and feed approvals, approved GM plants with introduced RNAi-based traits should be considered as not needing country-by-country rereview within the proposed globally harmonized risk assessment model.

### Retransformation of vegetative varieties

Vegetatively propagated plants such as banana, citrus, cassava, potato, and strawberry differ from sexually propagated or seed grown plants. For example, soybean and maize produce seed through sexual recombination (meiosis) resulting in progeny that are genetically and phenotypically different from their parents. Conversely, vegetatively propagated plants are genetically identical to their parent and have not undergone meiotic recombination or segregation. For this reason, vegetatively propagated plants are considered genetically stable (UPOV, 2002) and when plants such as potato are uniform, they can also be considered stable without need for additional stability testing (UPOV, 2004).

Simplot’s GM potato products provide an example where agency conclusions concerning the safety of a GM vegetatively propagated crop remained consistent after subsequent reviews (Pence et al., 2024). Simplot has 78 approved petitions for commercial GM potato events globally (Table 1). Of these submissions, the three traits in the pSIM1278 construct have been approved 74 times (Table 1). No hazards were identified in these independent reviews.

In recognition of this unnecessary strain on time and resources, some regulatory agencies have implemented retransformation policies to address regulatory redundancy and the low risk of subsequent events. The Canadian Food Inspection Agency’s Directive 94-08 “*Assessment Criteria for Determining Environmental Safety of Plants With Novel Traits*” (Section 5.4; CFIA, 2017) defines retransformation as “a transformation of a plant with the identical construct(s) as a previously authorized plant of the same species” and gives the developer the opportunity to notify the agency of the retransformed plant instead of undergoing the full determination process. The retransformed plant must meet three criteria: the traits are expressed in a similar range to the previously authorized plant; it is known, based on characterization, that the plant does not display any additional novel traits and is substantially equivalent to the previously authorized plant in terms of its use and safety; and novel food and feed requirements are met as appropriate. The CFIA’s notification process is an efficient and streamlined process that does not compromise the environmental safety of the product.

A similar approach is recommended for all approvals of new traits in vegetatively propagated plants. Experience supports extending the approval for one event to all subsequent events in the same crop with the same traits. This will reduce the regulatory applications for agencies and developers without decreasing the safety of cultivation and use of these GM plants. Retransformation of new events of vegetatively propagated GM plants would not need repeated risk assessments within the proposed globally harmonized risk assessment model.

### Gene insertions that mimic plant breeding—cisgenic plants

Cisgenic plants result from the introduction of genes from the same or closely related species and are often regulated under the same GM frameworks as transgenic plants. Schouten et al. (2006a) coined the term cisgenic plant nearly two decades ago and advocated for exemption of these plants from traditional GM regulations (Schouten et al., 2006b; 2006a; Jacobsen and Schouten, 2008).

The introduction of cisgenes through biotechnology has significant advantages compared to traditional breeding methods, especially for polyploid and vegetatively propagated plants. In potatoes, for example, true potato seed is not planted for commercial production because the genetic variability in the seed results in variable offspring with widely different characteristics and properties compared to the original parents. As a result, conventional plant breeding is not efficient for moving new traits into potatoes. To maintain the commercial quality traits that consumers, growers, and processors have come to expect from specific potato varieties, commercial potatoes are vegetatively propagated.

Maintaining genetic clones over years, decades, and even a century, as in the case of the Russet Burbank potato variety, comes at a cost. Pathogens, such as those causing late blight, often evolve rapidly and can overcome natural disease resistance in plants resulting in susceptibility to disease.

Although some wild and cultivated potato varieties possess naturally occurring plant resistance genes (R-genes), which provide resistance to late blight, breeding these traits into commercial potato varieties is time-consuming, labor-intensive, and often involves the transfer of undesirable traits. As an example, one attempt to breed the late blight R-gene *Rpi-blb2* from the wild potato species *Solanum bulbocastanum* into cultivated potato, took breeders 46 years (Hermsen, 1966; Hermsen and Ramanna, 1973; Haverkort et al., 2009). The resulting varieties, Bionica and Toluca, are used only on a limited scale because various agronomic and quality traits are not optimal for certain purposes (Jo et al., 2016).

Cisgenic approaches, by contrast, allow breeders to introduce desired genes from wild or crossable species into commercial varieties without disrupting other important agronomic or quality characteristics. More importantly, this can be accomplished in shorter time than the decades it takes for traditional breeding, while maintaining the desired traits of the variety. This approach has been used successfully to improve disease resistance in both vegetatively propagated plants including potato and apple (Vanblaere et al., 2011; Haesaert et al., 2015; Lazebnik et al., 2017) and polyploid plants including wheat and barley (Holme et al., 2012; Maltseva et al., 2021).

As our understanding of plant genetics and breeding advances, there is increasing recognition that regulatory oversight should prioritize the novelty and risk of traits rather than the technology used to introduce them. In particular, genetic insertions involving cisgenes should not be subjected to heightened regulatory scrutiny if those same genes feasibly could be introduced through conventional breeding methods. The European Food Safety Authority (EFSA) acknowledged that the introduction of cisgenes using biotechnology is at least as safe as conventional plant breeding (EFSA Panel on Genetically Modified Organisms, 2012). This approach is scientifically sound and represented by regulatory policy for cisgenic plants in agencies, such as HC, CFIA, U.S. Environmental Protection Agency (EPA), UK Department for Environment, Food and Rural Affairs (DEFRA), Food Standards Agency (FSA), and other national regulatory authorities (Health Canada, 2022a; CFIA, 2023; Environmental Protection Agency, 2023).

Health Canada and CFIA regulate novel food and plants with novel traits, respectively, emphasizing the novelty and potential environmental or health risks posed by the trait, regardless of how the trait was introduced and taking into account whether foreign DNA remains in the plant (Health Canada, 2022b; CFIA, 2023). These regulatory frameworks are intentionally process neutral. Importantly, when a cisgenic trait is introduced through biotechnology that could have otherwise been achieved via conventional breeding, and the trait itself is not novel in terms of food, feed, or environmental exposure, HC and CFIA may not consider the food, feed, or plant to be novel and will regulate the product as they would a conventionally bred plant.

In 2023, the United States EPA published a final rule exempting certain cisgenic plant incorporated protectants (PIPs) (Environmental Protection Agency, 2023). Under the Federal Insecticide, Fungicide, and Rodenticide Act, EPA regulates pest protection traits resulting from the introduction of genetic material into plants, which they refer to as PIPs. The 2023 rule exempts a PIP active ingredient if it is a characteristic of the population of sexually compatible plants, is created through genetic engineering from either an insertion of a native gene or a modification of an existing gene in the recipient plant, the substance is identical in sequence to the substance in the source plant, and the inserted regulatory regions are identical to those in the source plant (Environmental Protection Agency, 2023).

These examples illustrate the evolving perspective of regulatory agencies on cisgenes derived from sexually compatible species, however, more progress is needed. The EPA’s regulation maintains “genetic engineering” as the trigger for exercising its regulatory authority, focusing on the process used rather than the product resulting from the change. In addition, one factor that could inhibit developers from using this cisgene exemption is the presence of short segments of DNA used to introduce the gene of interest, such as *Agrobacterium* left and right border sequences, or short intervening sequences used during construct development. It can be argued that non-coding, non-expressed DNA sequences associated with cisgenes pose no additional risk and should be exempt from the EPA regulations.

It would be valuable for developers and farmers if more governments and regulatory authorities would categorize cisgenic traits as conventionally bred traits based on the evidence of the safety of these cisgenic changes in plants. This pivot would reflect a science-based approach that evaluates the risk of the product, and not the process by which it was developed, and would fit into a framework of global regulatory approvals, where traits based on cisgenes have been reviewed and demonstrated to be safe. Agencies like HC, the US EPA, CFIA and FSA have taken steps in the right direction. There is an opportunity now for regulatory authorities in other countries to revise regulatory policies that are not risk based, thus identifying cisgenic traits as low risk and good candidates for one global harmonized risk assessment.

## Proposed model for global risk assessments

**FIGURE 1:**
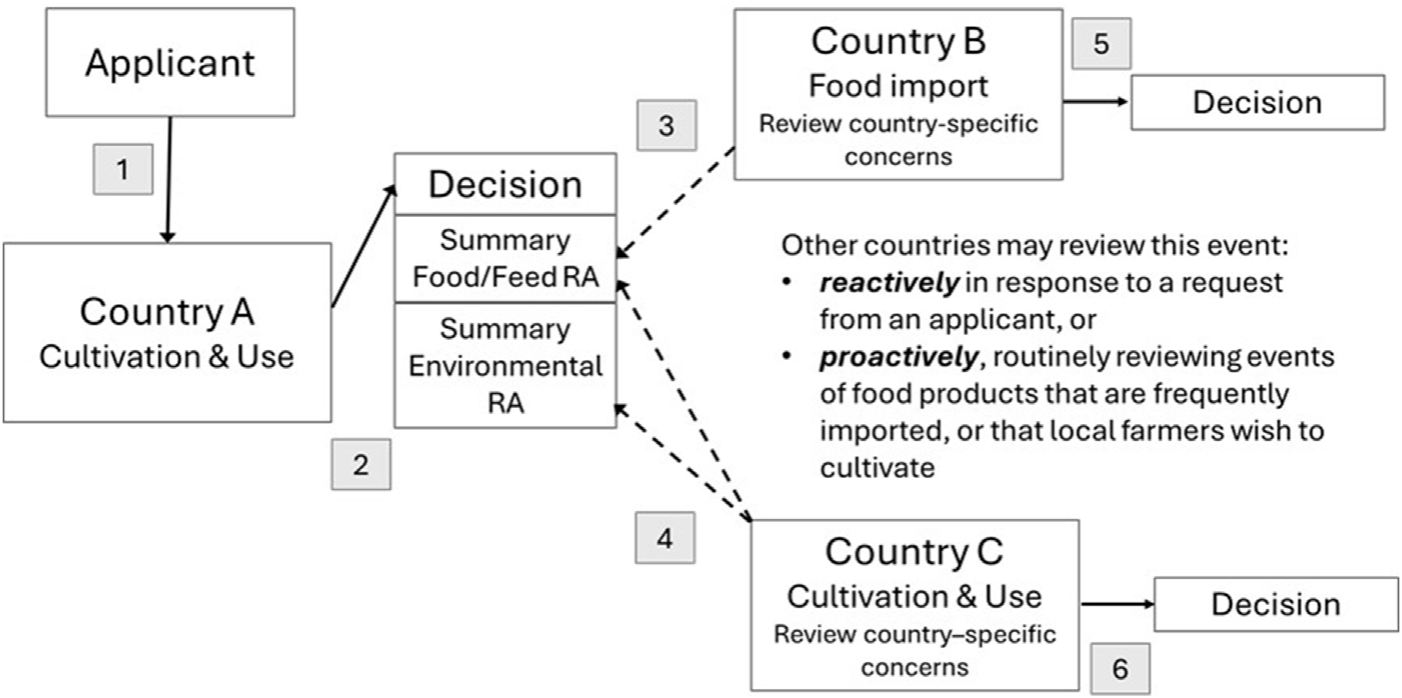
Proposed Model for Global Risk Assessments of GM Plants. Steps in the model are 1. Applicant applies for cultivation and use in country of first release, Country A. 2. Country A reviews the dossier and reaches a decision, posting risk assessment (RA) summaries on their website. 3. Food import Country B can access the food/feed RA summary from Country A and consider any additional hazards not already reviewed. 4. Country C, wishing to cultivate and use the GM plant, can access both food/feed and environmental RA summaries from Country A and consider any additional hazards not already reviewed. 5. Country B issues a decision on food safety. 6. Country C issues a decision on cultivation and use.

The proposed model for global risk assessments (Figure 1) is designed to minimize regulatory duplication while maintaining a high level of safety. This model maintains country sovereignty for decision making and encourages sharing of risk assessment summaries so that reviews in subsequent countries can focus on country specific potential hazards that were not previously assessed. In this model, after the first review and decision, other countries may review a GM plant reactively in response to a request from an applicant, or proactively by reviewing events for food products that are frequently imported or that local farmers wish to cultivate.

Countries may need to review their local regulatory policy to enable use of risk assessment summaries from other countries for national decisions. In some cases, country policy already lends itself to this process, for example, when regulation focuses on the safety of the product and not on the process used to develop it. In countries where there is a requirement for a formal applications to trigger a review, policies would need to be revised, and specific data requirements may need to be broadened to enable use of risk assessment summaries from other countries. The model does not stipulate what data are required, instead it relies on the outcome of a sound risk assessment using established risk assessment methodology.

Food and feed risks are universal, as are most environmental risks, however, some traits may raise concern unique to some countries. In this case, the national regulatory authority would identify the specific concern and outline a potential pathway to harm (Wolt et al., 2010). When using a global risk assessment summary, regulators use the biology of the conventional plant as the baseline for assessing risk. Once the biology of a plant is understood for the local environment, the impact of the added traits can be assessed to identify any new potential risk. This concern would be provided to the developer to address prior to decision making in that country.

Importantly, outside of regulations for pre-market review of GM plants, the safety of all food and all planting material is already regulated in countries. Implementing global GM plant regulatory processes does not impact the ability to review the safety of a product, nor detract from national public health regulations for food safety and plant pest regulations for environmental impact of conventional plants.

## Discussion

This paper considers the experience regulators have developed with safety reviews for GM plants in the last four decades and proposes a progressive change in how these plants are regulated internationally. The proposal is for one food and feed and one environmental risk assessment summary that will be available to all countries for national decision making. These two risk assessment summaries would be the product of the first country approval. While not all countries issue risk assessment summaries, enough countries make these available to enable the one review model to work.

The model proposed here expands on an earlier proposal for the identification of a core set of studies to support food and feed safety assessments globally (Waters et al., 2021). Using data from one country, these studies could be applied across all countries for risk assessment. These authors concur that problem formulation could be used to identify unique hazards on a country-by-country basis. Our experience, obtaining regulatory approvals for potatoes, suggests that not only are the data requirements between regulatory agencies already harmonized, but the first risk assessment with the harmonized data would be sufficient for all countries to reach national food and feed safety decisions.

Similarly, this model expands on an earlier proposal to simplify and modernize the data requirements for environmental risk assessment of GM plants (Anderson et al., 2021). There is already alignment on environmental protection goals across many countries, making the alignment of data requirements and data transportability across countries an easy step in harmonization of environmental risk assessments (Garcia-Alonso et al., 2014; Nakai et al., 2024). The model presented here suggests that the first environmental risk assessment is applicable to most countries for local decision making. Some countries may identify country-specific potential environmental hazards that need additional data and risk assessment, but no additional safety is achieved by repeating the review of the hazards already addressed in the initial assessment.

While regulatory cooperation and harmonization preserve national sovereignty, their implementation demands sustained political resolve. The AFSI, 2025, whitepaper for advancing regional cooperation on agricultural biotechnology regulation states “Regulatory cooperation and harmonization do not compromise domestic autonomy but do require political will to implement.” Several examples of harmonization across countries have emerged. Through the Health Canada-FSANZ Shared Assessment Process, applicants seeking food use of their biotech product for use in Canada, Australia, and New Zealand can submit an application to Health Canada and FSANZ. The food assessment is prepared by one of the agencies and then shared with the other. This risk assessment sharing is functioning and results in time and resource saving benefits for the agencies, improved efficiency in the authorization process, faster authorization times, and cost saving for the applicant. The Memorandum of Understanding, between Argentina, Brazil, Paraguay, and Uruguay, is another example of how countries can cooperate in regulatory assessments, while also maintaining domestic autonomy (Ministerio de Economia, 2025). As these examples evolve, they will serve as models for other countries and regions. Importantly, countries do not need MOUs to implement the model proposed here. Countries can make unilateral decisions to accept existing regulatory risk assessment summaries for GM plants they deem to have no additional risk for local conditions.

Reasons for a country to conduct further review would include country-specific information that raises a new potential hazard or an identified hazard in a new release environment, however these types of cases should be rare and based solely on traits that would pose identifiable and clearly documented risks. In particular, approved products that have been on the market for years, and which are cultivated safely in the environment, and consumed safely by humans and animals, likely would not require re-review.

The regulatory improvements suggested in this model also incorporate regulatory trends to extend approvals from one event to subsequent events with the same traits in the same crop, and to widen approvals for low risk traits. Evidence shows that approved RNAi-based crops and cisgenic crops pose no change to risk that is different from conventional, already-used food crops. Many regulatory agencies have recognized the safety of RNAi and cisgenes, especially when the traits could arise through traditional breeding. This alignment and a shift toward product-based, risk-proportionate oversight rather than process-based regulation, reflects current science and supports innovation. Similarly, reassessing vegetatively propagated crop varieties with the same trait or traits that have already been approved for food and environmental safety, has not identified new risks. The repeated reviews slow innovation, and result in unnecessary time and cost spent by both developers and governments.

### Practical considerations

There are several practical considerations that would contribute to a successful global risk assessment strategy for GM plants (Table 2). Global harmonization of risk assessment for GM plants is possible if the practical considerations described in Table 2 are implemented with a focus on process improvement and identified risk. Technology that has the potential to address global challenges of climate change and food security could be accessed more broadly if regulators spend less time reviewing products with proven low risk and demonstrated safety.

**TABLE 2:** Practical considerations for implementing one global risk assessment for a GM plant.

Consideration	Option
National sovereignty	Ensure that decisions to approve the use of a new GM plant remain a national obligation. In this model, the primary food and feed or environmental risk assessment objectively defines the safety of new GM plants and is used by national regulatory authorities to make national decisions for cultivation and use
First approval	Currently, cultivation approvals are submitted in the countries where the plants will be grown and food and feed submissions in countries that import products likely to contain the approved GM material. Under a one global risk assessment scenario developers are likely to submit the first application for a new GM plant to an agency that is science-based, functional, efficient, and cost effective
Separation of environmental safety and food and feed safety concerns during reviews	In our experience, countries that struggle to make efficient food and feed safety decisions often mix environmental safety into food and feed safety reviews (Koch et al., 2024). For efficient risk assessment, it is important to undertake review of food and feed safety separately from environmental safety. These reviews have different considerations, functions, and end points. As such, they should be considered separately. For example, a request for food and feed approval for imported nonviable food products does not need to consider issues like weediness and gene flow that might be important for a cultivation approval
Access to the primary risk assessment summary	Some agencies provide risk assessment summaries on their regulatory websites, making these available to other countries, such as trading partners, wishing to review the plants as they are approved. Agencies that produce publicly accessible risk assessment reports include the CFIA, FSANZ, European Food Safety Authority (EFSA), and the United States Department of Agriculture (USDA). Other regulatory agencies or countries can begin to think about ways to leverage these summaries in their own review and decision-making processes
Regulatory decision making	If, in reviewing a risk assessment summary from another country, an agency identifies a country specific potential environmental or food and feed hazard not considered in the first review, they could ask the developer for additional information on this specific hazard, without the needed to repeat the full environmental or food and feed biosafety review

### Benefits

The benefits of single global regulatory food, feed and environmental risk assessments are numerous (Table 3). For all regulatory agencies, access to global environmental or food and feed risk assessment summaries would reduce time required for reviews and the need for specialist knowledge. Using established international standards for food, feed and environmental risk assessments will ensure scientific and objective safety reviews that are universally applicable. National agencies would review the safety summaries and make decisions for local approvals after taking into consideration local conditions, priorities, and protection goals. Access to risk assessment summaries would allow countries to approve new GM plants proactively in response to national needs without receiving formal regulatory applications.

**TABLE 3:** Benefits of access to regulatory risk assessment summaries for GM plants.

Beneficiary	Benefit
Regulatory agencies	Reduce time required for reviews
	Focus on country-specific potential hazards
	Reduce the need for knowledge specialists
	Provide confidence that the reviews were conducted scientifically and objectively
	Make national decisions based on country specific protection goals
	Eliminate duplicate reviews of low-risk and familiar traits
Countries	Enable proactive national approvals linked to farmer, consumer, and local business needs without formal applications for GM plants Access to improved planting material will:
	• Improve sustainable food production
	• Mitigate food shortages
	• Build agricultural and horticultural economies
Developers	Eliminate the cost of multiple submissions to additional countries
	Redirect regulatory funding to product development
	Reduce time to market
	Eliminate duplicate reviews of low-risk and familiar traits
Public sector and small developers	Leverage regulatory experience in national agencies for faster approvals and lower regulatory costs
Farmers	Increased access to diverse planting material with desired traits and sustainable production benefits to protect farms and produce
Commerce	Strengthen agricultural and horticultural sectors of the economy
Consumers	Access to safe and affordable food
	Adoption and acceptance of GM foods and agriculture

For public sector and small developers, one environmental and one food and feed safety review would eliminate the cost of multiple submissions to additional countries. In our experience, this cost saving can be millions of dollars, funding that could be used on development and safety assessments for additional products, rather than on duplicated submissions and risk assessments that do not add to the safety of already approved products.

Long term benefits for farmers and consumers would be increased access to diverse planting material with desired traits. This access could help improve sustainable food production and mitigate food shortages around the world. In time, wider use of safe GM products could help to normalize the use of GM plants.

## Actionable recommendations

Actionable recommendations arising from this regulatory risk assessment proposal are listed in Table 4.

**TABLE 4:** Actionable recommendations for implementing global risk assessments.

Responsibility	Actionable recommendation
National regulatory agencies responsible for reviewing and approving GM plants	Based on the model, identify policy changes allowing implementation of a process to leverage the use of another country’s risk assessment summaries to reduce regulatory redundancy
	Make risk assessment summaries and decisions available on regulatory websites. These records of the hazards identified and assessed are key to enabling adoption of global risk assessments for GM plants
	In making decisions, consider widening the approval, where scientifically sound, to future varieties of the plant with the same traits. This is particularly true for RNAi traits and any retransformation of vegetatively propagated plants with the same traits
	Revise policy to treat GM plants with cisgenic traits as conventional varieties derived from plant breeding. Focus on regulating the product rather than regulating the technology used in development
National representatives of member countries at the United Nations Food and Agriculture Organization (FAO) and World Health Organization (WHO)	Work together to review and update the CODEX guidelines for Foods Derived from Modern Biotechnology (FAO/WHO, 2009). After more than 30 years, safety assessment guidance should be updated to align with the vast experience of regulators. Consider all the recommendations in this paper, the recommendation on unintended effects from the Canadian regulators (Schnell et al., 2015) and the recommendations on genetic stability in vegetatively propagated plants during clonal propagation (Pence et al., 2024)

## Conclusion

Decades of experience with risk assessments and approvals of GM plants have identified areas where regulatory efficiencies are possible without reducing the safety of GM products. Global access to environmental, food and feed risk assessment summaries from the first approval of a GM plant would remove the redundancy of numerous duplicated risk assessments in countries that subsequently review the plant for local cultivation or food import. With access to risk assessment summaries, countries can review these food, feed and environmental risk assessments and focus their risk assessment on unique potential hazards not previously reviewed. Maintaining national decision making, ensures that the final approval of GM plants remains the responsibility of each country. In addition, expanding approvals for GM plants with RNAi or cisgenic traits and to subsequent events of vegetatively propagated plants with the same traits, is supported by the low risk associated with these modifications. We encourage national regulatory agencies to implement this model which will reduce their reviewing time without compromising safety. This will provide benefits to developers by reducing regulatory redundancy, to farmers by facilitating increased access to improved planting material allowing more sustainable food production, and to consumers by providing access to high quality, affordable food.
